# Detection of Coconut Clusters Based on Occlusion Condition Using Attention-Guided Faster R-CNN for Robotic Harvesting

**DOI:** 10.3390/foods11233903

**Published:** 2022-12-03

**Authors:** L. G. Divyanth, Peeyush Soni, Chaitanya Madhaw Pareek, Rajendra Machavaram, Mohammad Nadimi, Jitendra Paliwal

**Affiliations:** 1 Department of Agricultural and Food Engineering, Indian Institute of Technology Kharagpur, Kharagpur 721302, India; 2 Department of Biosystems Engineering, University of Manitoba, Winnipeg, MB R3T 5V6, Canada

**Keywords:** machine vision, deep learning, object detection, coconut harvesting, occlusions, attention mechanism

## Abstract

Manual harvesting of coconuts is a highly risky and skill-demanding operation, and the population of people involved in coconut tree climbing has been steadily decreasing. Hence, with the evolution of tree-climbing robots and robotic end-effectors, the development of autonomous coconut harvesters with the help of machine vision technologies is of great interest to farmers. However, coconuts are very hard and experience high occlusions on the tree. Hence, accurate detection of coconut clusters based on their occlusion condition is necessary to plan the motion of the robotic end-effector. This study proposes a deep learning-based object detection Faster Regional-Convolutional Neural Network (Faster R-CNN) model to detect coconut clusters as non-occluded and leaf-occluded bunches. To improve identification accuracy, an attention mechanism was introduced into the Faster R-CNN model. The image dataset was acquired from a commercial coconut plantation during daylight under natural lighting conditions using a handheld digital single-lens reflex camera. The proposed model was trained, validated, and tested on 900 manually acquired and augmented images of tree crowns under different illumination conditions, backgrounds, and coconut varieties. On the test dataset, the overall mean average precision (mAP) and weighted mean intersection over union (wmIoU) attained by the model were 0.886 and 0.827, respectively, with average precision for detecting non-occluded and leaf-occluded coconut clusters as 0.912 and 0.883, respectively. The encouraging results provide the base to develop a complete vision system to determine the harvesting strategy and locate the cutting position on the coconut cluster.

## 1. Introduction

Coconut *(Cocos nucifera)* is one of the main cash crops grown throughout the year, predominantly along the coastal regions of Asia, South America, and the Pacific Islands. Coconut cultivation globally accounts for about 11.8 million hectares of agricultural land across 93 countries and nearly 63 million tonnes per year [1]. Among the Asian countries that contribute to almost 75% of the global production (52 million tonnes), Indonesia (17.1 million tonnes), Philippines (14.8 million tonnes), and India (14.7 million tonnes) account for more than 90% of the coconut production [2].

Coconut products have become an indispensable part of our day-to-day lives and for numerous industries in many parts of the world due to their high nutritional value and wide range of food and industrial products [1]. However, farmers are often trapped in financial losses despite increased demand for coconut products due to costly human labour and arduous work in many stages of its production. Manual harvesting of coconuts is one such operation that involves many operational risks and safety issues because of the tree’s height and hard trunk. Conventionally, coconuts are harvested by specially trained and experienced climbers who climb to the tree top and then cut the coconut bunches. This operation is hazardous, as falling from a tree due to slippage can result in severe irrecoverable physical damage and sometimes death [3]. Because of this high-risk manual harvesting operation, most young people are not interested in adopting it as an occupation despite decent wages. This scenario has led to an acute shortage of trained and skilled labour for this high-skill-demanding operation, especially during peak harvesting season. 

In order to mechanize this operation, various tree climbing devices have been proposed [4,5,6,7]. Abraham et al. [8] developed a remote-controlled coconut tree climbing robot in which the ground operator can regulate the locomotion and harvesting operations with the help of a camera mounted on a robot. As an improvement, Dubey et al. [9] proposed an autonomous climbing robot; however, its harvesting and spraying mechanisms needed to be manually controlled. 

With the rapid advances in artificial intelligence and robotics technologies over the past decade, autonomous vision-based robotic harvesting has emerged as a preferred approach for the selective harvesting of various fruits and vegetables [10,11]. In general, a typical vision-based fruit-harvesting robot includes two main subsystems: a machine-vision system and an end-effector system. The vision system detects and localizes the harvesting targets, and then the end-effector approaches these targets. The successful execution of the harvesting task requires accurate detection and localization of the harvesting targets [10,12]. Moreover, information about the robotic arm’s working environment is crucial for obstacle-free access to these targets. 

Thus, the effectiveness of robot end-effector manipulation depends greatly on the performance of the fruit detection algorithm. With the recent advancements in computational resources and the evolution of deep learning as a promising tool, researchers are increasingly relying on deep learning algorithms for developing vision-based harvesting systems [13,14,15]. Unlike traditional computer vision solutions that relied on identifying important features and tedious feature-engineering tasks [16,17,18], deep learning uses convolutional neural networks (CNNs) to automatically learn important features within training data, resulting in a less biased model. Deep learning algorithms have shown potential in many machine-vision operations applied to agriculture [19,20,21,22]. Numerous studies have employed CNN-based fruit detection models to support robotic harvesting, such as in the case of apples [23,24], tomatoes [25], strawberries [26], mangoes [27], kiwifruit [28], and blueberries [29]. 

In the case of the robotic harvesting of coconuts, the robotic end-effector could be damaged if the robot attempts to harvest occluded coconut clusters, which results in poor efficiency and economic loss. In recently developed coconut harvesting machines, the end-effector consists of a sharp circular high-speed rotating blade intended to cut the stalk of coconut bunches. For instance, Megalingam et al. [7] proposed a robotic arm with four degrees of freedom and a rotatory cutter end-effector. However, such an end-effector is very likely to damage the tree crown. Therefore, a crucial step for an automated harvesting system is to approach the coconut bunches via a path that avoids collision with the occlusions. For that purpose, the coconut bunches should be detected according to the occlusion condition. Thus, the objective of this study was to develop a deep learning object-detection model based on an improved Faster R-CNN algorithm to detect the coconut clusters as two classes: non-occluded coconuts and leaf-occluded coconuts.

## 2. Related Work

The performance of the fruit detection algorithm greatly determines the efficiency of autonomous harvesting. Over the past few years, there has been remarkable progress in developing smart fruit detection systems [13]. The conventional methods of fruit detection involve deriving the colour, texture, and morphology-based features present in the images [30,31]. However, they suffer from poor detection performance and robustness and fall short of the working needs of robotic harvesters. These image-processing-based algorithms are also prone to errors in unstructured backgrounds, occlusions, and different varieties of fruit (such as in the case of coconuts). In recent years, CNN-based algorithms have shown great advantages in fruit detection [32,33]. Many researchers have studied multi-class fruit identification in a variety of orchards for robotic harvesting [32]. Among various deep learning models, the Faster R-CNN family of networks has shown great potential for detecting objects of different sizes and aspect ratios, which makes them extensively useful for fruit detection [23,24,28,34,35,36]. 

In this regard, Fu et al. [23] used Faster R-CNN for detecting apples from RGB-D images of dense foliage orchards. They compared the performances of ZFNet and VGG16 networks for feature extraction, and the highest mean precision value of 0.89 was achieved by VGG16. The same networks as the feature extraction component of Faster R-CNN were compared for their result of multi-class apple fruit detection based on the occlusion condition [24]. Again, the VGG16 achieved the highest mean precision of 0.88 for the four-class detection at an average speed of 0.24 s per image. In another study, the Faster R-CNN model achieved the highest F1-score of 0.84 for sweet pepper detection [34]. Wan et al. proposed an improved Faster R-CNN network for the detection of apples, mangoes, and oranges [36]. Their model showcased a mean precision value of 0.86, which was higher than that of YOLOv3. Parvathi and Selvi [37] proposed a Faster R-CNN-based model to detect two main coconut maturity stages of consumer interest, namely, ripened and tender coconuts, under complex field conditions. The authors reported that the performance was better than other object-detection models, such as YOLOv3, R-FCN, and Single Shot Detector (SSD). Henceforth, motivated by results from these studies, we adopt the Faster R-CNN network for developing the machine-vision system for detecting coconuts based on their occlusion conditions.

Moreover, some previous studies have implemented an attention module to further optimize the deep learning model. The attention mechanism focuses more on the important features and reduces the huge number of intermediate features. For instance, a small CNN model (with 2.13 million parameters) with an attention mechanism and feature fusion module was designed to identify wheat ear diseases in field conditions [38]. The mentioned study achieved an accuracy of 94.1% and reduced the impact of complex backgrounds. The mechanism has also shown potential for the segmentation [39] and detection tasks [40]. The recent YOLOv4-tiny model was enhanced with adaptive feature fusion and attention modules to detect green peppers for developing a robotic picking system [41]. Notably, this model improved occluded and overlapped green pepper detection with an average precision of 95.1%, which was better than the performance of the algorithm tested without the attention mechanism. Hence, an attention module was additionally attached to the Faster R-CNN model to highlight and captivate the necessary features for coconuts detection.

## 3. Materials and Methods

### 3.1. Dataset and Software

#### 3.1.1. Image Acquisition

**Figure 1:**
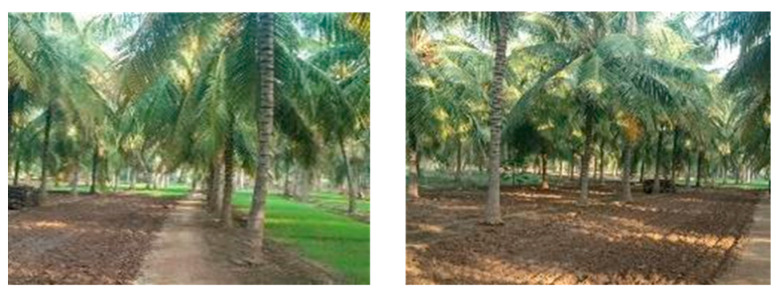
Commercial coconut plantation from where the dataset was collected.

The image data for this study were collected during a harvest season (July 2021) on a commercial coconut plantation in Western Tamil Nadu, India (Figure 1). The height of the coconut trees on the farm ranged from around 8 m to 20 m. The temperature and humidity ranged between 25–35 °C and 78–83%, respectively, with occasional clouds during the daytime. A total of 900 Red-Green-Blue (RGB) images of coconut tree crowns were acquired during daylight between 08:00 to 17:00 (morning to evening) under natural lighting conditions using a handheld digital single-lens reflex (DSLR) camera (Nikon-D3200, 40–60 mm lens, with a resolution of 300 × 300 dpi and 6016 × 4000 pixels). The experimenter stood on the ground near the tree trunk and used the camera to photograph the tree crown. Since the camera of the vision system in a coconut tree climbing robot would be facing towards the crown, the images collected in this study (the experimenter also points the camera upwards towards the tree crown) were analogous to the ones that would be captured by the system. Images were acquired standing at multiple locations around the tree so that different sections of the tree crown were covered. The path aside from every plantation row was used by the experimenter to move to subsequent trees.

**Figure 2:**
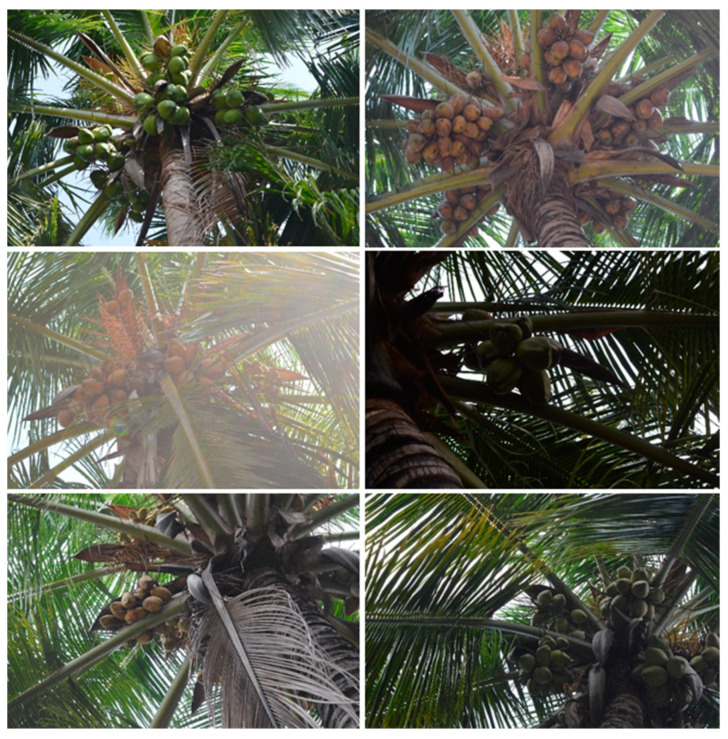
Sample images in the coconut dataset.

The dataset experienced variation in the illumination conditions when the sun angle changed, which could aid in the development of a more robust detection model. Because the ultimate motive of this study was to develop a machine vision platform for an automated coconut harvester (real-time application), the images were collected at different positions (elevation), angles, and lighting conditions (frontal, back, and scattered lighting) to ensure effective generalization of the model. The images also featured diverse conditions associated with the background, shadows, and tree crown orientation. The images were resized to 512 × 512 pixels since the original size was too large and demanded high computational resources and time for detection. A few images from the dataset are presented in Figure 2. The dataset can be accessed at https://data.mendeley.com/datasets/w4t73tvrf8.

A Windows 10-based system with *MATLAB^®^* R2021b (Mathworks Inc., Natick, MA, USA) software was used for the analysis. The Image Processing Toolbox, Statistics and Machine Learning Toolbox, and Deep Learning Toolbox were additionally installed into the *MATLAB^®^* software.

#### 3.1.2. Coconut Classes and Ground Truth Preparation

**Figure 3:**
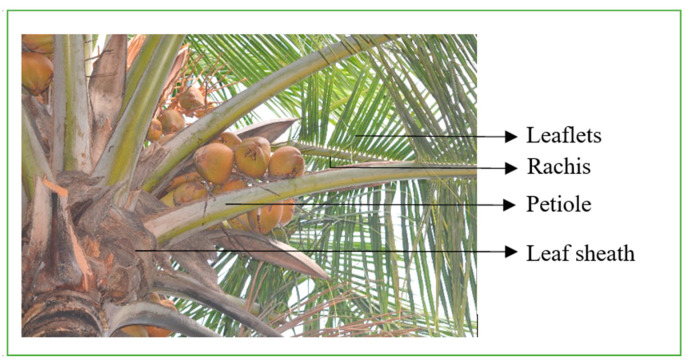
Occluded coconuts and structure of coconut leaf.

**Figure 4:**
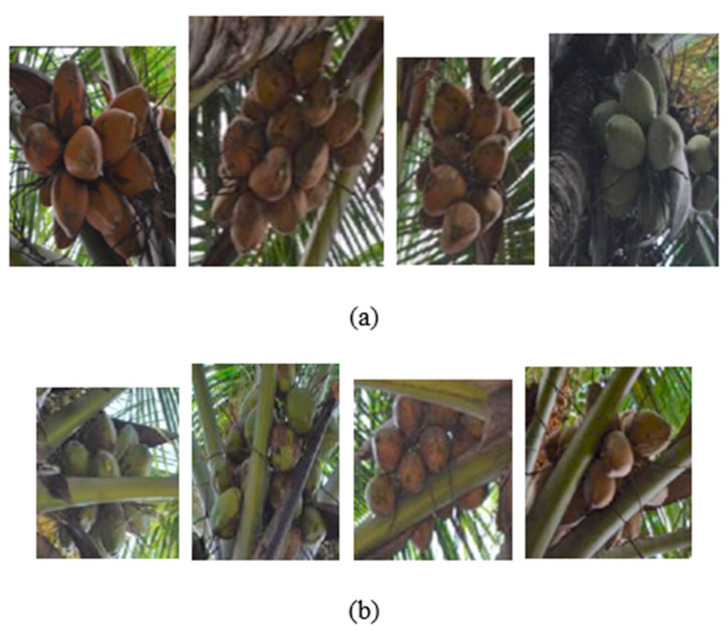
Coconuts categorized into two classes based on the condition of occlusion: (**a**) non-occluded coconuts and (**b**) leaf-occluded coconuts.

Unlike apples and berries, coconuts cannot be harvested through common robotic approaches like picking or shaking mechanisms due to their hard nature. The region of impact on the coconut cluster for robotic harvesting is often occluded by the petiole, cushion, leaflets, or other parts of the tree crown (Figure 3). Hence, the coconut clusters were categorized into two classes based on occlusion (Figure 4). The first ‘non-occluded coconuts’ class represents the independent coconut clusters that can be harvested directly with priority. The second ‘leaf-occluded coconuts’ class refers to the coconut clusters occluded by the various portions of the coconut leaf. 

**Figure 5:**
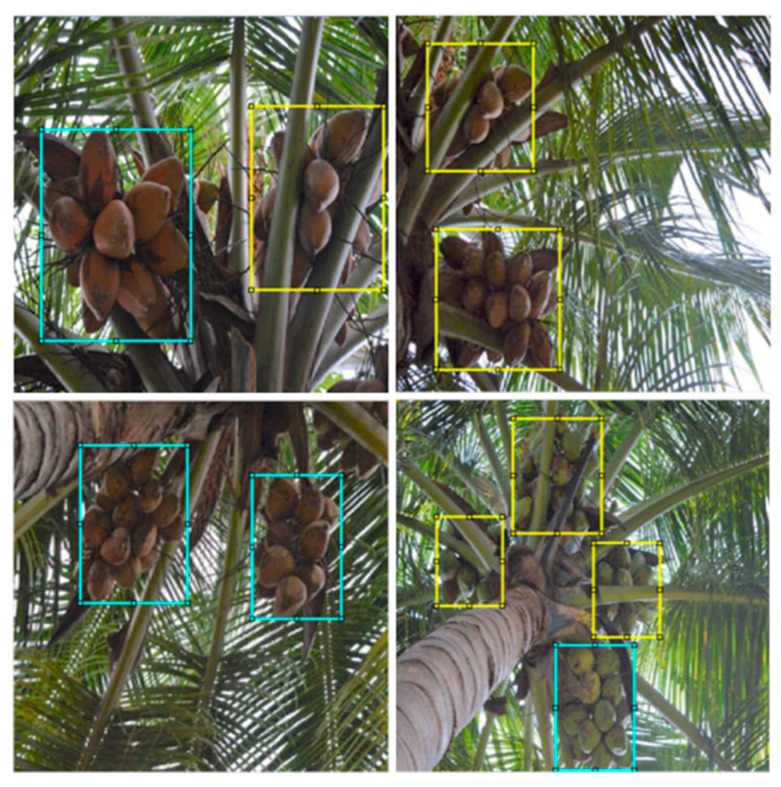
Rectangular annotations of coconut targets for creating ground truth. The blue and yellow bounding boxes refer to the non-occluded (NOC) and leaf-occluded (LOC) coconut targets, respectively.

The coconut clusters were annotated into two classes with rectangular bounding boxes (Figure 5). All images were manually annotated using the interactive *MATLAB^®^ Image Labeler* app. The following abbreviations/class names were used to indicate the classes (which will be followed in the rest of the article): ‘NOC’ for non-occluded coconuts, and ‘LOC’ for leaf-occluded coconuts. 

#### 3.1.3. Image Augmentation

To increase the size of the dataset and prevent the model from overfitting, geometry- and intensity-based image augmentations were additionally performed. Image geometry-based augmentation techniques that were adopted include random rotations at 90°, 180°, and 270° as well as horizontal and vertical flipping. While performing the above geometry transformations, a small code strip was written in the *MATLAB^®^* environment to automatically calibrate the bounding box coordinates of the transformed images from the original image’s bounding box annotations. Furthermore, image intensity transformations included brightness variation with propositional coefficients of 0.8, 0.9, 1.1, and 1.2 as well as histogram equalization after converting the RGB images to their HSV (Hue-Saturation-Value) equivalents. The blurring of images is a potential problem in vision-based harvesting systems due to the camera’s movement. To make the detection network more adaptable to such real conditions, a Gaussian filter with a standard deviation value of two was applied to the images as another augmentation strategy for artificial blurring. 

Thus, the size of the dataset was increased by 12 times (from 900 to 10,800 sample images) through data augmentation, which was randomly separated as follows: 6800 images for training the model; 2000 images for validation; and 2000 images for testing purposes. The class-wise distribution of coconut clusters in the dataset is presented in Table 1.

### 3.2. Deep Learning Network Architecture

**Figure 6:**
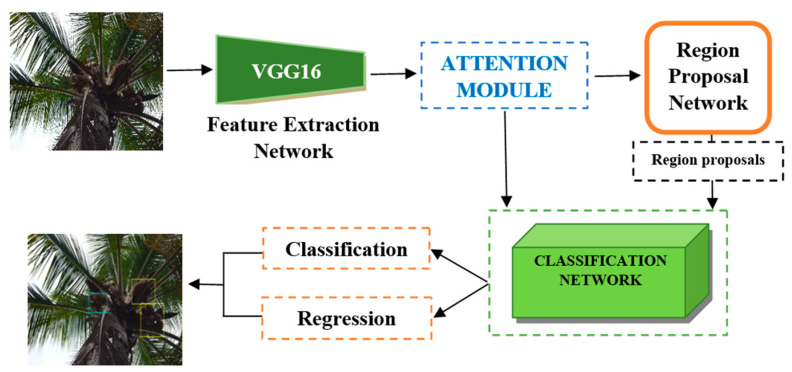
Illustration of the architecture of the proposed coconut cluster detection system based on Faster R-CNN with VGG16 and the attention module.

The architecture of the proposed coconut detection model is detailed in this section. The structure of the improved Faster R-CNN model is illustrated in Figure 6, which consists of a features extraction network, a Region Proposal Network (RPN), and a final classification network. To improve the quality of features derived from the feature extraction network, an attention mechanism was affixed to this feature extraction network to concentrate on the informational channels more than the less important channels. 

#### 3.2.1. Feature Extraction Network and Attention Module

In the proposed model, the VGG16 network was incorporated as the backbone feature extraction network. The network comprises four maximum-pooling layers that divide the thirteen convolutional layers into five groups. These pooling layers help extract a broad range of feature maps from different levels of the network. To enhance the feature representation, this was followed by an attention module (before feeding the feature maps into the RPN) that amended and improved the feature maps and provoked a better quality of region proposals by emphasizing the descriptive feature channels. 

In the attention module, a global average pooling operation is initially performed on the input feature map to convert them into a channel descriptor. The descriptor is then passed into two successive fully connected layers, which were respectively modulated by the ReLU and sigmoid function. This arrangement aids in gaining the properties of a channel from a global perspective and handling channel-wise interdependencies. The output from the second fully connected layer (modulated by sigmoid function) accords an attention descriptor β, whose values emulate the importance of the respective channels. If $X\in {\mathbb{R}}^{H\times W\times C}$ denotes the input features to the attention module, the H×W dimensional feature maps were compressed to a channel descriptor $z\in {\mathbb{R}}^{1\times C}$ by the dense layer as in Equation (1).

(1) $$z_{c}=\frac{1}{H\times W}\;\sum_{i=1}^{H}\sum_{j=1}^{W}X_{c}\left(i,j\right)\;$$

**Figure 7:**
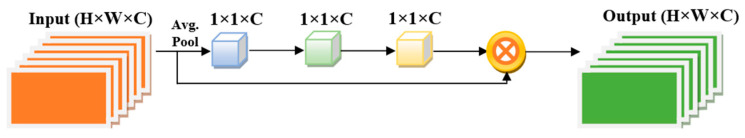
Illustration of the attention module employed in the proposed detection model.

The descriptor z is converted into the probabilistic attention descriptor $\left(\beta \in {\mathbb{R}}^{1\times 1\times C}\right)$ by the sigmoid operation. Thus, the input feature channel maps are recalibrated in accordance with the attention descriptor by multiplying this channel-by-channel (Equation (2)) to generate informative feature maps with more emphasis.

(2) $$Y_{Cn}=F_{hadamard}\left(X,\beta \right)=\beta ⊙X$$

where $F_{hadamard}$ denotes the Hadamard product (channel-wise element-wise multiplication). The attention module used in the coconut detector is presented in Figure 7. The final feature maps are fed into the RPN for region proposal.

#### 3.2.2. Region Proposal Network and Classification Network

A typical region-based object detection CNN is based on the following steps: (i) identify the region that might contain the object (called the region proposals), (ii) extract the features from the proposed regions, and (iii) classify the objects and adjust the bounding box. The RPN, which is also a fully convolutional network, is used to create the region proposals using the features from the VGG16 feature extractor. The region of interest pooling operation (ROI-pooling) is carried out to transform these region proposals (which do not have a uniform size) into the same size. In the ROI-pooling layer, the non-uniform region proposals are divided into a fixed dimension, say $N_{w}\times N_{h}$ sub-blocks, and a max-pooling operation is carried out in each sub-block. This constructs a feature map of fixed sizes with the most salient features. These fixed-size bounding box guesses are called anchors, which are uniformly placed throughout the image to capture the aspect ratio of target objects. The typical dimensions (height and width) of anchor boxes $\left(N_{w}\times N_{h}\right)$ were chosen based on the size of the coconut clusters in the images. 

The classification network leverages the RPN’s proposals and the feature maps from the attention module as the inputs. This network consisted of two convolution layers and two fully connected layers followed by two branches (a region classification branch and a regression branch) for fine-tuning the anchor box coordinates. Based on the scores and predictions of the classification layer, the positions of the plausible anchor boxes can be traced back from the output of the CNN back to the input image. Once the position of an anchor box is mapped, the localization errors (between the ground truth bounding box and the predicted tiled anchor box) are fixed. Through the regression branch, the detector learns the offsets to be applied to the anchor box to adjust its position and size. 

### 3.3. Network Training

The attention-guided Faster R-CNN network with VGG16 as the feature extraction network was trained for the task of coconut cluster detection. The fully convolutional RPN was optimized using the back-propagation algorithm and mini-batch gradient descent, with a loss function comprising the sum of the classification and bounding box regression losses. The hyperparameters set during the training process are provided in Table 2. The *MATLAB^®^* application was run on Acer Nitro 5 Intel Core i5 9th Generation Laptop (32 GB/1 TB HDD/Windows 10 Home/GTX 1650 Graphics).

### 3.4. Performance Evaluation Metrics

The performance of the detection model was evaluated using the weighted mean Intersection-over-Union (wmIoU) and mean Average Precision (mAP) metrics. 

#### 3.4.1. Weighted Mean Intersection-over-Union

**Figure 8:**
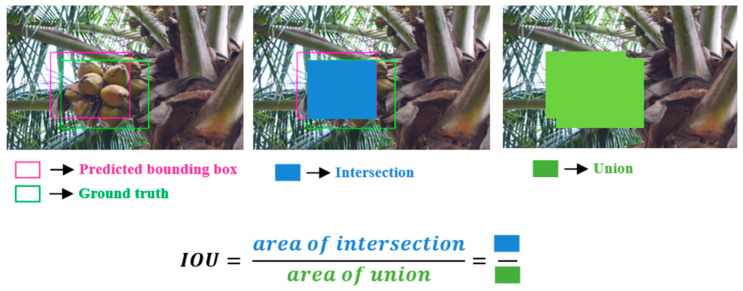
Illustration of the intersection over union (IoU) evaluation metric.

Intersection-over-Union (IoU), or the Jaccard similarity coefficient or Jaccard index, is a measure of the overlap between the ground truth and predicted bounding boxes. The IoU can be determined via the ratio of the area of overlap to the area of the union of two bounding boxes (Figure 8). Predicted bounding boxes that have heavily overlapped with the ground truth have a higher IoU value compared to ones with less overlap. If $A_{predicted}$ denotes the predicted bounding box and $A_{groundtruth}$ is the ground truth, then IoU is mathematically expressed as Equation (3).

(3) $$IoU=\frac{A_{predicted}\;\cap \;A_{groundtruth}\;}{A_{predicted}\;\cup \;A_{groundtruth}}$$

Intuitively, the mean IoU (mIoU) for an image is the average of IoU values of the predicted bounding box with the ground truth for each class in the image. In this work, to reduce the biasing effect of IoU for dominating classes, the mIoU was weighted with the number of objects in each class $\left(X_{c}\right)$ for all images in the dataset, called the weighted mean IoU (wmIoU). The wmIoU can be expressed in terms of mIoU for N images and C classes as in Equation (4).

(4) $$wmIoU=\frac{1}{N\times C}\times \sum_{c=1}^{C}X_{c}^{-1}\sum_{n=1}^{N}mIoU$$

#### 3.4.2. Mean Average Precision

In this study, a detection was considered a positive prediction if the IoU was greater than 0.5. With that, all detections were classified as true positive (*TP*), false positive (*FP*), true negative (*TN*), or false negative (*FN*). Thus, precision (*P*) and recall (*R*) were defined as:

(5) $$Precision\;\left(P\right)=\frac{TP}{TP+FP}$$

(6) $$Recall\;\left(R\right)=\frac{TP}{TP+FN}$$

The global performance of a network to detect the target object is determined by its Average Precision (AP), defined as the area under the precision–recall curve (Equation (7)), which is obtained by plotting P and R in the vertical and horizontal axes, respectively. If *AP_c_* represents the AP of a particular class *c*, then the mean AP (mAP) is given as the average precision of all classes (Equation (8)). Hence, the greater the value of *AP_c_*, the better the results of the coconut detection system. The value of c represents the classes considered in the experiment: NOC (*c* = 1) and LOC (*c* = 2).

(7) $$AP_{c}=\int_{0}^{1}P\left(R_{c}\right)\;dR_{c}$$

(8) $$mAP=\frac{1}{C}\sum_{c=1}^{C}AP_{c}$$

## 4. Results and Discussion

**Figure 9:**
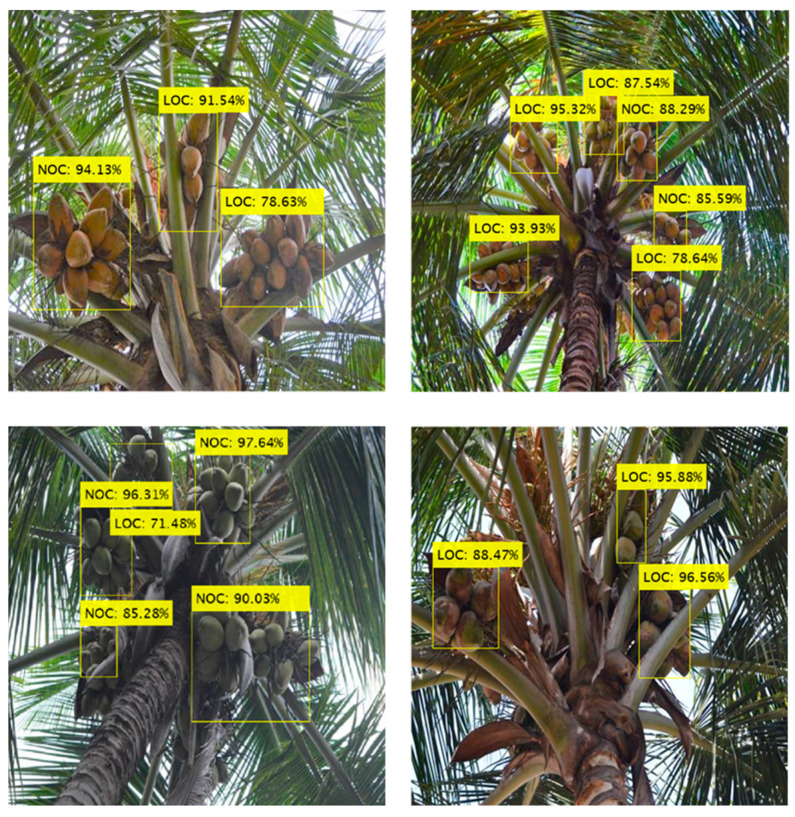
Detection of coconut clusters using the proposed model. The labels show the detected class (NOC: Non-Occluded Coconuts; LOC: Leaf-Occluded Coconuts) and confidence score.

In order to verify the performance of the attention-guided Faster R-CNN model for the detection of coconut clusters, 2000 images were used to test the model. The mAP and wmIoU values for the test data were 0.88 and 0.83, respectively. The class-wise detection results on test and validation sets are presented in Table 3, and example images are illustrated in Figure 9. Since NOCs are completely visible, they were easier to be detected with a higher mIoU (0.90) and AP rate (0.91). The LOCs were also identified with better precision, but the mIoU value (0.80) was relatively low. This shows that although the LOC clusters were detected with high precision, the regression loss due to localization of the bounding boxes was high, thus resulting in reduced IoU measure (which indicates the proximity of predicted and ground truth bounding boxes). The model was also able to effectively detect coconut clusters occluded by many leaves (Figure 9). 

**Figure 10:**
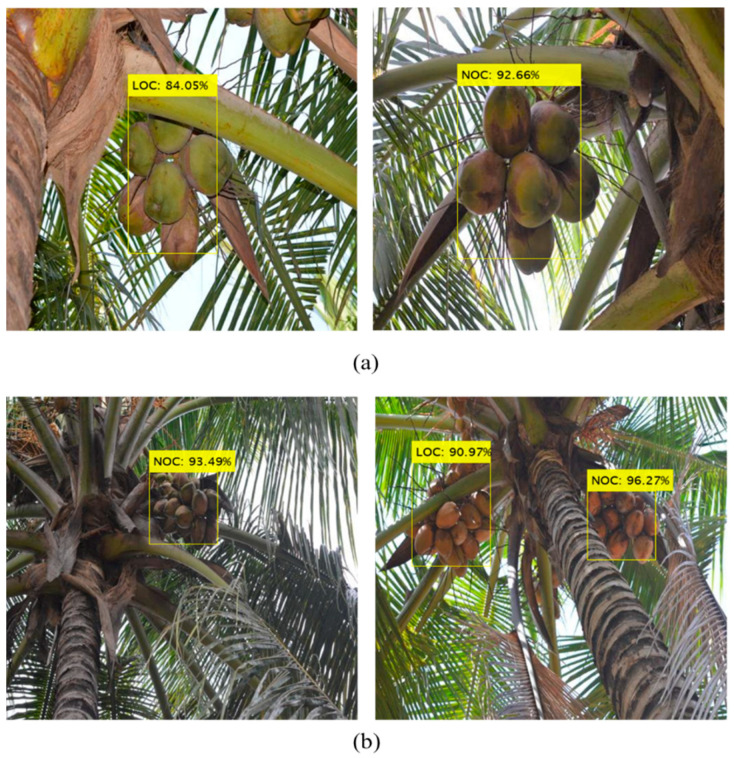
Coconut detection result concerning tree crown proximity: (**a**) near to the tree crown and (**b**) farther from the tree crown.

The model took 0.774 s on average to detect coconut clusters in images of size 512 × 512 pixels, which was deemed adequate for developing a real-time vision-based harvester. The size of the model’s trained weights was 548 MB, comparable to other Faster R-CNN-based models in the literature designed for real-time fruit detection [13,24]. Because the coconut detection system would potentially be deployed in a tree climbing robot, the size and number of clusters in the field of view will differ with its movement on the tree trunk. The size would be larger, and fewer coconuts would be visible when the vision system is near the tree crown, hence making it easier to detect. However, more clusters will be present in the field of view when the robot is farther. Therefore, the model’s performance was analyzed for test images that were acquired closer to the tree crown and farther away from it (Figure 10). The images were considered to be taken farther from the crown when more than approximately 20% of the length of the tree was captured in the image. Inspection of the images showed that bounding boxes detected for close images had a better prediction of the ground truth than the farther images that had intensive coconut clusters. Nevertheless, the result for closer images is more significant since harvesting is performed at the time when the robot is near the tree crown. Moreover, it is to be noted that the anchor box size used by the model can have a deliberate impact on the recognition of coconuts of different sizes and the box’s aspect ratio. 

**Figure 11:**
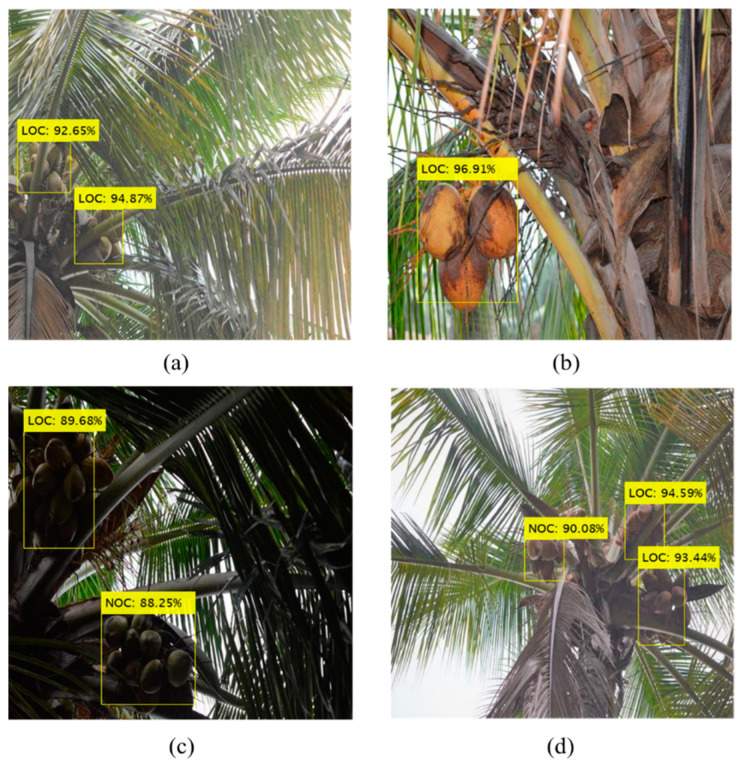
Detection of coconut clusters under different illumination: (**a**,**b**) direct sunlight, and (**c**,**d**) backlight conditions.

The results imply that the model can facilitate the detection of different numbers of coconut clusters from the image captured from any position on the tree trunk. Including an attention mechanism provided higher weight to the features of coconuts, thus improving the ability to recognize coconuts with a range of occlusions. As shown in Figure 11, the proposed method was suitable for images with different illumination conditions. In addition, the detection results were impressive for images collected under direct sunlight (Figure 11a,b) and backlight (Figure 11c) conditions. The method was also applicable for the detection of poorly illuminated coconut clusters under strongly backlit conditions (Figure 11d). However, the manual analysis showed that the IoU value was lower for detection in images with intensive coconut clusters under backlighting. Unlike the detection of small fruit such as apples and strawberries, which experienced poor performance due to shadows and similarities with backgrounds [26,42], coconut detection under such conditions appears relatively easier due to its large size and unique shape among the objects in the image’s field of view. 

Practically, the colour of coconuts differs greatly in a plantation due to the accommodation of many varieties, which comprise shades of green, yellow, orange, and brown. It was observed through the experiment that this did not have any impact on the prediction performance, meaning that the principal features utilized for coconut detection were shape- and texture-based. Additionally, very accurate detection of the coconut clusters when their edges are occluded is problematic since their sizes and shape vary greatly (unlike fruit such as apples, whose shape can be very closely determined even when partially occluded). In real-time, the vision system may also capture images without any coconuts during the robot’s operation. To test the validity of the model in this case, ten tree crown images that did not contain any coconut were derived from internet sources. As expected, no predictions were reported by the model in these images. 

**Figure 12:**
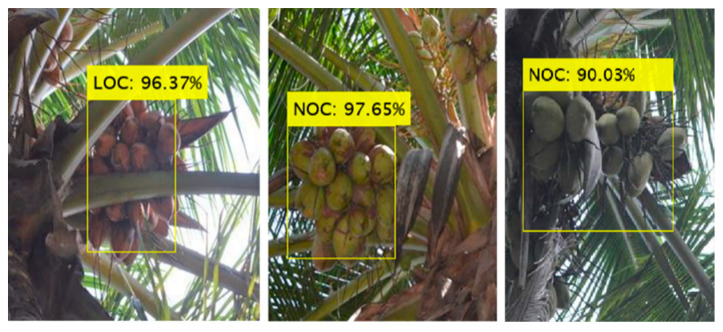
Misdetection of coconut-occluded coconut clusters; two clusters were detected as a single cluster of coconuts.

Since the features of coconuts belonging to different clusters in the same tree are very similar, identifying and differentiating the coconuts of two bunches when one occluded another was challenging (Figure 12). Although considerable detection of the coconut cluster causing the occlusion was possible, the model faced difficulty in placing the prediction box coordinates of the occluded coconut cluster. 

Overall, this study statistically analyzed the performance of a deep learning approach for coconut detection to assist the robot in planning the harvesting strategy based on occlusion conditions. The proposed model proved effective in detecting the coconut clusters into two classes with a high precision rate (Table 3). Many previous studies have stressed the potential of the attention mechanism in Faster R-CNN for improving the result as well as reducing the prediction time [43,44,45]. In the case of leaf-occlusion, either a special path must be adapted for the end-effector to reach the coconut bunch, or the leaf must be cut down before proceeding to harvest in order to free the cluster from obstruction. In that case, if many leaves occlude a cluster, more research should be carried out in the future to locate the position of leaves as well for efficient harvesting. With good results obtained through the attention-guided Faster R-CNN model for the detection of a complete coconut bunch, further investigation on model development is intended to be carried out for localizing and estimating the position of a cutting point in the cluster. 

Multi-class detection based on occlusion conditions for harvesting has been widely studied for other fruits, including apples [24,42], tomatoes [46], cereal grains [47,48], and citrus fruits [49]. Similar to the proposed model, Gao et al. [24] used Faster R-CNN with VGG-16 as a feature extraction network for detecting apples and classifying them into four classes based on occlusion (non-, leaf-, branch/wire-, and fruit-occluded). The model achieved mAP of 0.88 and AP of 0.91, 0.90, 0.86, and 0.85 for the respective classes. In a similar study, Mu et al. [46] used R-CNN with Resnet-101 to detect occluded tomatoes and achieved an mAP of 0.88. There have been no previously reported studies on real-time coconut detection for harvesting or other robotic applications. Hence, it is difficult to compare the results of this study with others. To the best of our knowledge, only Parvathi and Selvi [37] have worked on the development of a deep learning-based coconut detection system for maturity stage identification. They tested four algorithms, namely, Faster R-CNN, SSD, YOLOv3, and FCN, and concluded that the results achieved by Faster R-CNN (mAP = 0.89) were significantly better than the other models. Importantly, the maturity identification approach can be combined with occlusion condition analysis to give a better perception of the vision system for decisive coconut harvesting. However, such a model demands more classes to be comprehended. Another interesting topic for future research is the performance of similar state-of-the-art deep learning models in robotic systems for other applications such as grading agri-food products [50,51,52,53,54,55,56,57,58], monitoring crop diseases [59,60,61], and assessing crop growth and yield [62,63,64,65,66].

## 5. Conclusions

In this study, a deep learning model based on state-of-the-art Faster R-CNN with VGG-16 was proposed for detecting coconuts based on their occlusion condition. The analysis showed that the model achieved high precision and speed for identifying non-occluded and leaf-occluded coconuts, which can evade potential damage to the tree and robot end-effector during robotic harvesting. The attention mechanism was introduced into the Faster R-CNN model to improve the ability of the network to identify occluded coconuts. The model achieved mAP of 0.88 and wmIoU of 0.83 for coconut detection in the two classes, and the speed was 0.77 s per image. The proposed method offers valuable information in planning the end-effector movement for coconut harvesting safely and efficiently. 

## Figures and Tables

**Table 1:** Class-wise distribution of coconut clusters in the training, validation, and test datasets after performing image augmentation.

Class	Number of Coconut Clusters			Total Number of Coconut Clusters in the Class (in Complete Dataset)
	Training	Validation	Test	
Non-occluded coconut clusters	7674	2220	2310	12,204
Leaf-occluded coconut clusters	11,250	2856	2814	16,920
Total	18,924	5076	5124	29,124

**Table 2:** Some hyperparameters set during training of the model.

Hyperparameter	Value
Optimizer	Stochastic gradient descent with momentum (sgdm)
Initial learn rate	0.001
Maximum epochs	1000
Mini batch size	32
Learn rate drop factor	0.0005
Learn rate drop period	10
Momentum	0.9

**Table 3:** Class-wise results for coconut cluster detection using attention-guided Faster R-CNN.

	mIoU		Average Precision (AP)	
	Validation	Test	Validation	Test
Non-occluded coconuts	0.906	0.895	0.924	0.912
Leaf-occluded coconuts	0.812	0.807	0.899	0.883

## Data Availability

The data presented in this study are available on request from the corresponding author.
